# Effect of ductus deferens lavage on the time to achieve azoospermia in patients undergoing vasectomy

**DOI:** 10.6061/clinics/2018/e504

**Published:** 2018-09-24

**Authors:** Francisco Barros de Oliveira, Vadelias Xavier Pereira, Fernando Rocha Oliveira, Luiz Carlos de Abreu, Blanca Elena Guerrero Daboin, Alex Rey Norberto, Luiz Vinicius de Alcantara Sousa, Luis Fernando B Tavares, Sidney Glina

**Affiliations:** I Programa de Pos-graduacao, Faculdade de Medicina do ABC (FMABC), Santo Andre, SP, BR; II Laboratorio de Escrita Cientifica, Faculdade de Medicina do ABC (FMABC), Santo Andre, SP, BR; III Disciplina de Urologia, Faculdade de Medicina do ABC (FMABC), Santo Andre, SP, BR; IV Programa de Pos-graduacao, Faculdade de Medicina FMUSP, Sao Paulo, SP, BR; V Laboratorio de Epidemiologia e Analise de Dados, Faculdade de Medicina do ABC (FMABC), Santo Andre, SP, BR

**Keywords:** Vasectomy, Sterilization, Irrigation, Contraception

## Abstract

**OBJECTIVE:**

To evaluate the effect of normal saline lavage of the distal vas deferens ampulla in patients undergoing vasectomy on the time to achieve azoospermia.

**METHODS:**

A prospective randomized study of 60 men divided into two groups, group lavage (GL, n=30) in which distal vas deferens ampulla lavage was performed with 10 ml of normal saline during the vasectomy, and group without lavage (GWL, n=30) in which control patients received only a vasectomy. The patients provided sperm for semen analysis at the 5^th^, 10^th^, 15^th^, 20^th^ and 25^th^ ejaculations.

**RESULTS:**

Fifteen participants in GL and 16 in GWL, for a total of 31 patients, were excluded due to not completing the control spermiogram. The tests carried out at the five ejaculations showed immobile spermatozoa in 40 and 85.71%, 66.67 and 78.57%, 93.33 and 85.71%, 86.67 and 71.43%, and 93.33 and 85.71% of participants in GL and GWL, respectively.

**CONCLUSION:**

Vas deferens duct lavage with 10 ml of normal saline during vasectomy did not decrease the time required to achieve postoperative azoospermia.

## INTRODUCTION

Vasectomy is the most widely used contraception method owing to its effectiveness, low complexity and cost-effectiveness. Approximately 40 to 60 million men worldwide have been sterilized, and approximately 500,000 surgeries are performed annually. Vasectomy accounts for 5% to 10% of contraceptive use among all available methods 1-3.

Despite the effectiveness of vasectomy, the desired endpoint of azoospermia and consequent infertility is not achieved immediately. These events occur after a period of 90 to 120 days in most men due to residual spermatozoa located distally to the ligature 4,6.

Precautions to prevent pregnancy should be maintained until the spermiogram performed in the weeks following the surgical procedure confirm the absence of spermatozoa or their immobility, parameters used to authorize sexual intercourse without other contraceptive methods 2,3.

The time period and number of ejaculations required to achieve azoospermia have been subjects of many debates. Factors associated with the time to azoospermia include patient age and the number of ejaculations. The interval for azoospermia is shorter if two ejaculations occur per week for younger patients and one occurs per week for those over 40 years. Patients who ejaculate more than three times in 7 days present earlier with the absence of spermatozoa in the ejaculate, regardless of age 7-9.

An alternative method to decrease the time to azoospermia is lavage of the distal vas deferens ampulla. Different solutions, including those with and without spermicidal activity, have been used for this technique, and the volume has ranged from 2.5 to 50 ml 4,5.

The objective of irrigation is to utilize mechanical propulsion to empty the vas deferens content by immobilizing and lysing spermatozoa 10.

Most studies that tested irrigation of the vas deferens used distilled water and saline 4,10.

Other investigated irrigators include Euflavine hydrochloride, Nitrofurazone, Xylocaine, chlorhexidine, mercury phenyl nitrate, Diltiazem, methylene blue and Kystosol (5% sorbitol solution plus 0.25% acetic acid) 11-17.

The results of irrigation of the vas deferens at the time of vasectomy are not consistent, which has made it a topic of much discussion 9,11,13,18.

Irrigation of the vas deferens with distilled water immobilizes and lyses spermatozoa by osmosis, and several authors have tested volumes between 5 and 50 ml. A study performed with two groups of patients, those who underwent vasectomy without irrigation (n=87) and those who underwent vasectomy with irrigation (n=76), obtained a mean time to the absence of spermatozoa of 26.4 and 28.6 weeks, respectively, with no significant difference between groups. In the 40th week, the persistence of spermatozoa was 22% among patients who did not receive irrigation and 26% among those who did undergo irrigation 19.

The use of 0.9% saline solution at volumes ranging from 20 to 50 ml for irrigation of the vas deferens ampulla resulted in 8.92, 8.65 and 2.17 thousand spermatozoa/ml in spermiograms after 2, 6 and 12 weeks, respectively, whereas the corresponding values were 2.8, 1.3 and 0.2 × 10^6^ spermatozoa/ml in patients who did not receive irrigation. There were no statistically significant differences between techniques 4,5.

A study comparing patients who underwent vasectomy with irrigation with distilled water or 9 g/L saline solution after 16 weeks showed azoospermia in 100% of men (n=42) that underwent irrigation with distilled water, in 88.1% of men (n=37) irrigated with saline solution and in 26.2% of men (n=11) who did not undergo irrigation. There was a significant difference between the distilled water and saline solution groups 20.

Euflavine causes mutations in DNA and RNA and has spermicidal activity. Studies of vas deferens irrigation have compared 2 to 5 ml of Euflavine at a 1/100 or 1/1000 dilution with 5 ml of distilled water. The results showed complete elimination of spermatozoa after a mean of 11 ejaculations for distilled water and of 5.5 ejaculations for Euflavine 11.

Aqueous chlorhexidine crosses the spermatic membrane and alters its integrity, leading to cell lysis. A previous study evaluated three groups of patients: 1, vasectomy only; 2, vasectomy and irrigation with 5 ml of distilled water; and 3, vasectomy and irrigation with 5 ml of chlorhexidine at a 1/5000 dilution. The spermiogram results in the sixth week showed 59%, 28% and 7% persistence of immobile spermatozoa in groups 1, 2, and 3, respectively 14.

Nitrofurazone inactivates enzymes involved in cellular energy generation. Irrigation with 3 ml of 0.1% nitrofurazone led to 90% sperm immobility in the spermiogram of the second ejaculation 12.

Xylocaine inhibits depolarization and suppresses neural activity. The use of 10 ml of 2% xylocaine for vas deferens lavage in 1000 patients resulted in 86% immobile spermatozoa on the fourth day after surgery and 100% immobility in the sixth week 13.

Mercury phenyl nitrate, which exerts spermicidal activity by abrasion, was used at a 2% concentration for vas deferens irrigation at volumes of 2.5 and 5.0 ml. After 14 days, 35 of 48 patients showed azoospermia or immobile spermatozoa 15.

Kystosol is a dehydrating and abrasive solution; 20 ml of Kystosol was used for vas deferens irrigation, and patients who had undergone vasectomy without irrigation (n=70) or with irrigation (n=20) were compared. Spermiograms 2 weeks after surgery showed immobile spermatozoa in 2% of the individuals without irrigation and in 25% of those with irrigation. Two months later, these percentages were 44% and 90%, respectively. After 3 months, the azoospermia rate was 91% in non-irrigated group and 100% in the group that underwent Kystosol irrigation 17.

Studies have assessed different substances at varying volumes, but the results are controversial, and it is unclear whether irrigation should be incorporated into the vasectomy procedure to accelerate the time to achieve azoospermia. Therefore, we considered it justifiable to carry out this study.

The aim of this study was to evaluate the effect of ductus deferens lavage on the time required to achieve azoospermia in patients who underwent vasectomy.

## METHODS

This study is a prospective, randomized trial. Randomization was conducted in the waiting room preceding surgery. Data on the participants were placed inside small envelopes in a box, giving each participant the same chance of being in the vasectomy group with lavage (GL) or the vasectomy group without lavage (GWL).

The selected patients were enrolled in the Family Planning Program of the municipalities of Itupeva and Ibiúna, São Paulo, and all participants had been approved for a vasectomy. Patient approval was obtained after they were interviewed and evaluated by a psychologist and nurses working within the program, and patients were informed of all available contraceptive methods.

This project was approved by the Research Ethics Committee of the School of Medicine of ABC under protocol number 077365/2013.

The inclusion criteria were full civil capacity, over 25 years of age or at least two living children, and the completion of a 60-day period between the manifestation of the desire to undergo a vasectomy and the surgery to ensure sufficient time to reflect upon alternative contraceptive options.

Patients who did not agree to control spermiograms; those with scrotal sack damage, other trauma or neoplasia; and those with a prior failed vasectomy were excluded from the study. Data were collected from May 2015 until February 2016.

Initially, 60 individuals were included, but 31 patients were excluded after surgery (15 in GL and 16 in GWL) as they failed to undergo a control spermiogram, even though they had previously agreed to do so. Therefore, the final study sample included 29 patients who were divided into two groups: 15 participants in GL and 14 in GWL.

### Surgical technique

For GWL, the established vasectomy technique was used (resection and ligation of the vas deferens). The operation was conducted in a surgical center under standard local anesthesia (5 ml of 2% liquid Xylocaine without a vasoconstrictor).

A 2×2 cm area of the wall of the anterolateral and superior scrotal hemi-sac was anesthetized, starting with the right and then proceeding with the left. A 1-cm incision was made along the anatomical planes for exposure, and 2-cm resections were introduced in the right and left deferens.

Double ligatures were made in the proximal and distal segments using cotton thread (n°. 2.0). Hemostasis was reviewed, and access sutures were used.

For the vasectomy procedure in GL, the protocol followed the same steps as described above but also included lavage of the vas deferens. Lavage was performed by catheterization of the distal segment of each channel using a nontoxic, plastic 10 cc disposable syringe and one of two polyethylene catheters: Becton Dickinson Angiocath 22 G × 1 (0.9 × 25 mm; flow rate 28 ml/min) and 24 G × 0.7 (0.7 × 19 mm; flow rate 17 ml/min). The catheter was chosen according to the length of the vas deferens segment (varied from 2 to 3 mm) and was connected from the syringe to the catheter. The connection was digitally fixed. The patient was informed when irrigation was initiated, and irrigation was performed under moderate and gradual pressure to avoid the development of lesions. The average time for irrigation was 60 s, which corresponded with a slower rate than the catheter flow so that discomfort and the sensation of needing to urinate generated by the passage of liquid could be controlled by the study participant. Irrigation was performed with 10 ml of a 0.9% saline solution.

After irrigation, the catheters in the segments were gently removed, and the respective ligations were made. Hemostasis was reviewed, and access sutures were used.

### Spermiogram

After the vasectomy, the patients returned on postoperative day 7 and were instructed to perform a total of five spermiograms at predetermined moments, namely, the 5th, 10th, 15th, 20th and 25th ejaculations.

Participants received a sterile, wide-mouthed, sealed glass container to be opened at the time of ejaculation. The participants were taken to an isolated laboratory room where, after washing their hands, they were requested to collect a semen sample through masturbation and then immediately deliver the sample for analysis.

All samples were analyzed by the same laboratory technique 21 involving a Neubauer chamber for cell counting and optical microscopy at 200X magnification. For each sample, 25 fields within 16 smaller fields, for a total of 400 fields, were counted to verify the presence or absence of spermatozoa.

### Statistical analysis

For quantitative variables, descriptive statistics were used to describe and summarize the data. These included an assessment of the normal distribution and variability of the data, as well as the mean and standard deviation.

The Shapiro-Wilk test was used to verify the normal distribution of the data. To compare age and the mean time between the first and last exams between groups, Student's t-test and the Mann-Whitney test were applied according to the normality of the data. The chi-square test was used to compare groups based on the frequencies of successes and failures of individuals at each examination.

For this study, the significance level was set at 5%. All statistical analyses were conducted using SPSS version 22.

## RESULTS

Table 1 presents the mean patient age and the mean time between the first and last spermiograms in GL and GWL. No statistically significant differences in any variable were observed between groups.

In every spermiogram analyzed from the 5th ejaculation following vasectomy with or without irrigation onward, either azoospermia or the presence of immobile spermatozoa was recorded. Table 2 shows the number of patients in GL and GWL who produced samples without spermatozoa or with immobile spermatozoa. There was a significant difference between groups for the 5^th^ ejaculation only, for which 12 patients in GWL and six in GL showed azoospermia (*p*=0.011).

Spermiograms of two (13.33%) and four (28.57%) individuals from GL and GWL, respectively, showed residual spermatozoa after none had been observed in the previous examination.

**Figure 1:**
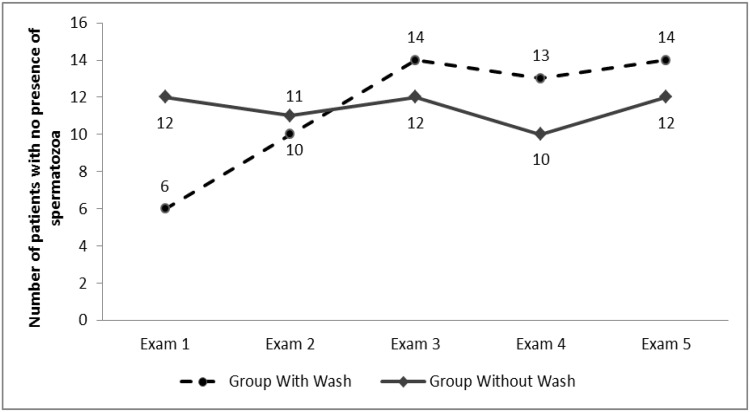
Number of individuals who presented with azoospermia at each exam.

When the frequencies of azoospermia among individuals in each group were compared according to examination number, there was an increased frequency of azoospermia in GL compared to GWL at each exam (Figure 1). Only exam 1 showed a statistically significant difference that favored GWL, as shown in Table 2.

## DISCUSSION

The study describes the effect of distal vas deferens lavage on the time to achieve sterilization in patients undergoing vasectomy. The literature includes reports on research in the United States, England, Italy, and India, but similar studies have not been reported in Latin American countries, especially in Brazil.

Sterility does not occur immediately following the procedure. Residual spermatozoa are ejaculated for weeks or months, independent of technique 18.

The time required to achieve sterility ranges from the third to the fourth month after surgery, and an estimated 20 ejaculations are required 16,17.

Vas deferens irrigation using distilled water was compared with that using saline solution, and significant differences between groups were found in regard to the azoospermia rate at week 8 (38.1% *versus* 0%), week 12 (100% *versus* 30.9%) and week 16 (100% *versus* 88.1%) 20.

A study involving 906 patients found a difference according to technique and concluded that vas deferens lavage accelerates azoospermia from 20% to 30% 22. Therefore, such lavage offers the option to avoid the longer-term use of alternative contraceptive methods.

The results of this research would likely be more impressive if the number of participants was higher, which would enable additional analyses to associate irrigation of the distal vas deferens ampulla with the absence of spermatozoa and earlier sterilization.

This work employed five exams at standardized times, namely, the 5^th^, 10^th^, 15^th^, 20^th^ and 25^th^ ejaculations after surgery. The mean time to azoospermia was 56.2 days (SD 32.12 days) in GL and 80.14 days (SD 61.1 days) in GWL, as shown in Table 1.

A statistically significant difference between groups was observed in the first exam, in which a larger number of individuals in GWL showed a lack of spermatozoa. However, this result could have been affected by self-reporting of the number of ejaculations before each exam, although the two groups received the same guidelines and requests for the number of spermiograms. The time between ejaculations and laboratory attendance were independent variables that may have generated inequality under predetermined conditions.

In regard to studies that have assessed the use of irrigation, different definitions of postoperative sterility have been used. Studies generally define intervention success as the absence of spermatozoa in two consecutive samples, even if they are present in later analyses.

Other definitions for azoospermia do not include the number of residual spermatozoa but are based on an evaluation of immobility alone or on the results of two consecutive tests, even though one study reported the presence of 10,000 up to 100,000 immobile spermatozoa 23.

In this research, success was defined as azoospermia. When spermatozoa were found, they were immobile, as reported in other publications 19. This finding has been reported in physiological studies on the content of the vas deferens following vasectomy 24,25.

Among vasectomy patients, 0.3% to 13% present with mobile spermatozoa in the first spermiogram after vasectomy 26, indicating that technical error or recanalization may have occurred. Knowledge about immotile spermatozoa is limited, as it remains unclear whether they can lead to pregnancy or if their presence is a sign of permeability of the vas deferens ampulla 27.

The persistence of immobile spermatozoa is a known phenomenon 27 that was seen in this work.

Philp et al. did not report any cases of gestation in the 2% of men that had persistent spermatozoa (<10,000/ml) 23.

Davies et al. reported no gestation after azoospermia in their series, and sperm remained in 150 men (<10,000/ml) at a follow-up of 3 years 28.

Edwards and Farlow achieved infertility in 200 individuals with persistent immobile sperm, of which 30 patients had counts of 500,000/ml and two had counts of 1,000,000/ml 29.

Spermatozoa were observed in this study, as shown in Table 2, but they were immobile, as reported in other studies 16,19,24.

Eisner et al. observed persistence for up to 70 days 30.

This study observed persistence until the 25th ejaculation: 13.33% persistence in GL and 28.57% in GWL.

Several centers have reported that persistence ranges from 0.8% to 2.4% 31.

Dirk and Edwards reported persistence rates of 33% and 42%, respectively, and the time to achieve azoospermia was 12 and 14 weeks, respectively, with a follow-up of 10 months 27.

O'Brien et al. reported the reappearance of spermatozoa 12 months after vasectomy with a count of <10,000/ml 32.

The reappearance of immobile spermatozoa is not expected after the patient has achieved azoospermia. Such reappearance occurred in six patients in this study, which may be explained by the slow release of male gametes from the seminal vesicle and the abdominal deferent ductus 28,32, as well as individual differences in the number of spermatozoa stored in these anatomical segments 33.

Samples produced at the 10^th^, 15^th^, 20^th^ and 25^th^ ejaculations showed a lower sperm count. The first exam at the 5th ejaculation was the exception, possibly due to the initial mechanical mobilization of spermatozoa by the saline flow.

Various results of the vasectomy lavage technique have been presented within the literature. A review by Cook et al. in 2007 argued that the divergent results are likely attributable to differences between protocols in relation to the quantity and type of solution used to perform vas deferens lavage, as well as to the surgical technique used 7.

The study results indicate that vas deferens lavage during the surgical procedure is a valid option because of its low cost, complexity and risk to the patient.

Similar to reports in other studies, the limitation of this study was the loss of participants over the postoperative follow-up period, usually related to the fact that the surgical act had already led to sterility despite study literature stating the necessity of multiple exams. This limitation can be overcome only by the inclusion of a large number of participants because of voluntary study protocol adherence.

The technique of vas deferens lavage with 10 ml of saline solution during vasectomy did not affect the time to achieve postoperative azoospermia.

## AUTHOR CONTRIBUTIONS

Abreu LC and Glina S participated in the design of the study. Oliveira FB carried out the study. Sousa LV and Oliveira FR analyzed the data. Daboin BE, Norberto AR, Tavares LF, Pereira VX and Oliveira FB drafted the manuscript.

## Figures and Tables

**Table 1:** Average patient age and mean time between the first and last spermiograms.

	Group with Lavage	Group without Lavage	
	Mean (SD) (n=15)	Mean (SD) (n=14)	p*
Age (years)	39.8 (8.53)	37.57 (5.93)	0.498
Time between the first and last tests (days)	56.2 (32.12)	80.14 (61.1)	0.370

SD, Standard deviation; p*, Mann-Whitney or t test.

**Table 2:** Comparison between the number of patients who showed an absence of spermatozoa or the presence of immobile spermatozoa.

	Vasectomy Technique				
	Group with Lavage		Group without Lavage		
	Without spermatozoa n (%)	With immobile spermatozoa n (%)	Without spermatozoa n (%)	With immobile spermatozoa n (%)	p*
Exam 1	6 (40)	9 (60)	12 (85.71)	2 (14.29)	0.011*
Exam 2	10 (66.67)	5 (33.33)	11 (78.57)	3 (21.43)	0.474
Exam 3	14 (93.33)	1 (6.67)	12 (85.71)	2 (14.29)	0.501
Exam 4	13 (86.67)	2 (13.33)	10 (71.43)	4 (28.57)	0.311
Exam 5	14 (93.33)	1 (6.67)	12 (85.71)	2 (14.29)	0.501

* Chi-square test
